# Kontaktlinsenassoziierte oberflächliche stromale Keratitis durch *Paecilomyces lilacinus*

**DOI:** 10.1007/s00347-020-01087-5

**Published:** 2020-03-24

**Authors:** L. Hübner, T. Tourtas, J. Weller

**Affiliations:** Universitätsaugenklinik Erlangen, FAU Erlangen-Nürnberg, Schwabachanlage 6, 91054 Erlangen, Deutschland

**Keywords:** Kontaktlinsenassoziierte Keratitis, Pilzkeratitis, Oberflächliches HH-Infiltrat, Voriconazol AT, Hornhautscraping, Contact lens-associated keratitis, Fungal keratitis, Superficial corneal infiltrate, Voriconazole eye drops, Epithelial debridement

## Abstract

Wir sahen 2 Kontaktlinsenträger mit Keratitiden durch *Paecilomyces lilacinus*. Die Besonderheit der Fälle liegt in der oberflächlichen Lage der Infiltrate im oberen Stroma ohne Infiltration in die Tiefe. Der Erregernachweis erfolgte durch eine Abrasio bzw. Hornhautscraping. Die mykotische Keratitis konnte in beiden Fällen durch eine intensive topische Therapie mit Voriconazol beherrscht werden. Bis auf eine EDTA-Touchierung und Amnionmembrantransplantation in 1 Fall waren keine weiteren chirurgischen Eingriffe erforderlich.

## Abstract (en)

This article presents two cases of contact lens wearers with keratitis caused by* Paecilomyces lilacinus*. The special feature of these cases was the superficial manifestation of the infection in the upper stromal layer without infiltration into deeper layers. The pathogen was identified following epithelial debridement and corneal scraping. In both cases the fungal keratitis could be successfully managed by intensive topical treatment with voriconazole. Except for an epithelial debridement with EDTA chelation and an amniotic membrane transplantation in one case, no further surgical interventions were necessary.

## Fall 1

### Anamnese

Eine 53-jährige Frau berichtete über eine seit 5 Tagen bestehende Rötung des rechten Auges mit Epiphora und starkem Fremdkörpergefühl. Vor 7 Monaten wurde eine kontaktlinsenassoziierte Keratitis am selben Auge erfolgreich konservativ behandelt (extern, daher keine weiteren Informationen); weiche Monatskontaktlinsen wurden anamnestisch inzwischen wieder getragen. Die Patientin war immunkompetent, ohne Allgemeinerkrankungen in der Anamnese.

### Befunde

**Figure 1:**
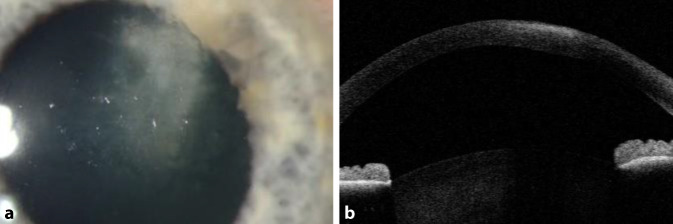

In der ophthalmologischen Untersuchung zeigte sich eine Erosio corneae (3 × 3,5 mm) mit aufgeworfenem Epithel an den Rändern ohne Infiltrat oder Vorderkammerreiz (Abb. 1a). Die Epithelirregularität und Erosio wurden zunächst auf einen Kontaktlinsentrageschaden zurückgeführt. Der Verdacht auf eine infektiöse Keratitis bestand initial nicht, da kein Infiltrat vorlag. Die Behandlung erfolgte lokal antibiotisch (Ofloxacin) und pflegend. Am 3. Tag sahen wir ein oberflächliches Hornhautinfiltrat (2,4 × 1,7 mm, Abb. 1b) mit gräulichem irregulärem Epithel, sodass wir eine fokale Abrasio corneae durchführten. Das Abradat wurde zur mikrobiologischen Untersuchung eingesendet, da das „dirty epithelium“ nun an eine infektiöse Ursache, z. B. Akanthamöben, denken ließ.

Bei Erstvorstellung bei uns war der Visus 0,4 mit der eigenen Korrektur. Am Partnerauge sahen wir einen reizfreien Befund mit klaren brechenden Medien und einem bestkorrigierten Visus von 1,0.

### Diagnose

In der Mikroskopie wurden keine Mikroorganismen nachgewiesen. In der Kultur konnte nach 2 Tagen das Wachstum von Schimmelpilzen beobachtet werden, die weitere Klassifikation ergab *Paecilomyces lilacinus*.

### Therapie und Verlauf

Nach Erhalt des kulturellen Befundes leiteten wir eine kalkulierte antimykotische Therapie mit Voriconazol 1 % Augentropfen (AT) stündlich ein, mit Zyklopentolat wurde zudem die Iris ruhiggestellt.

Unter oben genannten Therapie zeigte sich eine Besserung des Lokalbefundes. Vier Tage nach Erhalt der kulturellen Befunde lagen uns die Ergebnisse der Resistenztestung vor: Der Erreger war sensibel gegen Voriconazol und resistent gegen Amphotericin B, folglich konnte die Monotherapie mit Voriconazol 1 % AT stündlich fortgeführt werden.

**Figure 2:**
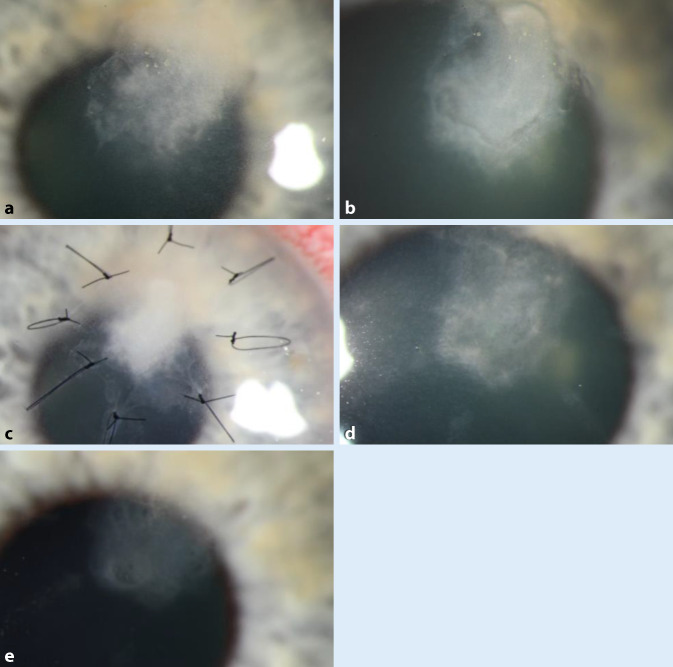

Aufgrund eines persistierenden Epitheldefekts entschlossen wir uns am 12. stationären Tag zur Aufnähung eines Amnionmembrantransplantatpatches (AMT-Patches). Am 17. Tag zeigte sich die Membran in Auflösung, wir sahen eine zunehmende Abgrenzung des Infiltrats mit einer Erosio corneae und oberflächlichen Verkalkungen (Abb. 2). Es folgte ein Hornhautscraping mit EDTA-Touchierung, da die Kalzifikationen die Epithelialisierung beeinträchtigten. Die Lokaltherapie mit Voriconazol 1 % AT stündlich wurde fortgesetzt. Der Epithelschluss fand schließlich am 24. Tag statt, das Infiltrat (2,0 × 1,3 mm) zeigte sich abgegrenzt und in Vernarbung übergehend. Die Patientin konnte unter Lokaltherapie mit Voriconazol 1 % AT 2‑stündlich und lokaler Pflege in die ambulante Weiterbehandlung entlassen werden. Voriconazol wurde langsam ausgeschlichen, insgesamt über einen Zeitraum von 3 Monaten appliziert. In der abschließenden Kontrolluntersuchung nach 7 Monaten zeigte sich eine inaktive oberflächliche Hornhautnarbe mit stromaler Verdünnung ohne Hinweis auf ein Rezidiv und einem Visus von 0,63 mit eigener Korrektur.

## Fall 2

### Anamnese

Ein 21-jähriger Mann stellte sich erstmalig mit seit 3 Wochen bestehendem Fremdkörpergefühl und Schmerzen am linken Auge vor. Der immunkompetente, allgemein gesunde Patient berichtete uns über das Tragen weicher Monatslinsen. Die aktuellen Linsen seien seit etwa 2 Wochen für ca. 8 h täglich unter regelmäßiger Kontaktlinsenhygiene verwendet worden. Vom niedergelassenen Augenarzt war zuerst mit Dexamethason/Gentamicin-Augentropfen für 2 Wochen, anschließend mit Aciclovir-Augensalbe (AS) und Prednisolonacetat AT – zum Zeitpunkt der Erstvorstellung seit 5 Tagen – vorbehandelt worden.

### Befunde

**Figure 3:**
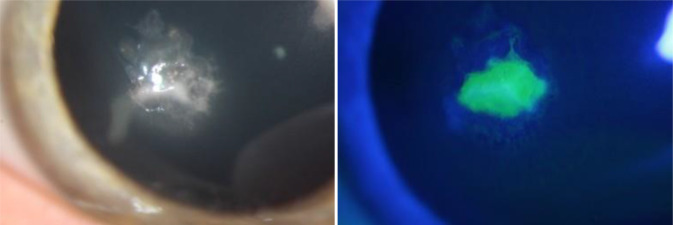

Im oberen Stroma waren ein irreguläres Hornhautinfiltrat mit Ausläufern, eine Erosio corneae (3,0 × 2,8 mm) sowie eine reizarme Vorderkammer auffällig (Abb. 3). Bereits bei Erstvorstellung in der Klinik (2 Wochen nach Therapiebeginn extern) wurde ein Hornhautscraping durchgeführt. Unter der initialen kalkulierten antibiotischen Lokaltherapie vor Erhalt des Erregernachweises mit Cefuroxim 5 % AT und Tobramycin 1,5 % AT zeigte sich eine leichte Besserung des Lokalbefundes; das Infiltrat grenzte sich ab, die Erosio und der Vorderkammerreiz zeigten sich rückläufig.

Der Visus mit der eigenen Korrektur war bei Erstvorstellung 0,5. Das Partnerauge stellte sich reizfrei dar mit klaren brechenden Medien und einem bestkorrigierten Visus von 1,0.

### Diagnose

Auch in diesem Fall blieb die Mikroskopie ohne Erregernachweis, kulturell wurde *Paecilomyces lilacinus* nachgewiesen.

### Therapie und Verlauf

Nach Erhalt des kulturellen Befundes leiteten wir eine kalkulierte antimykotische Therapie mit Voriconazol 1 % AT stündlich und Zyklopentolat ein. Aufgrund der initialen Besserung des Lokalbefundes unter der antibiotischen Lokaltherapie behielten wir eine zusätzliche antibiotische Abdeckung mit Cefuroxim 5 %/Tobramycin 1,5 % AT 3‑mal täglich bei. Die Resistenztestung zeigte schließlich eine Sensibilität gegen Voriconazol.

**Figure 4:**
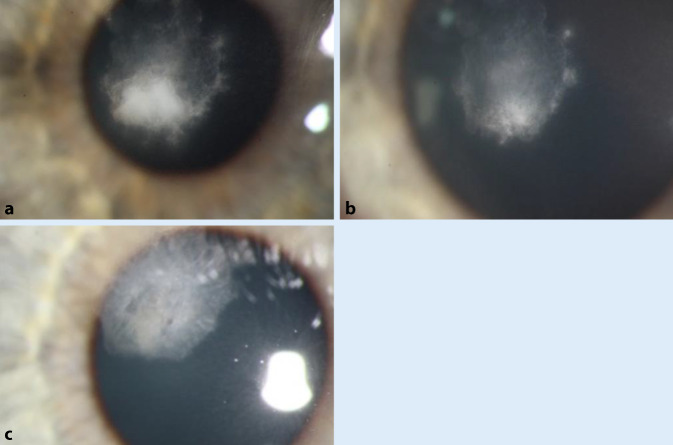

Trotz weiterer Abgrenzung des Infiltrats unter der antimykotischen Therapie führten wir bei zunehmenden weißlichen Ablagerungen über dem Infiltrat am 3. Tag eine Abrasio corneae durch (Abb. 4). Im weiteren Verlauf sahen wir ein geschlossenes Hornhautepithel sowie eine zunehmende Abgrenzung des Infiltrats, sodass wir den Patienten in die ambulante Weiterbehandlung entlassen konnten. Die Anwendung der Voriconazol 1 % AT wurde über einen Zeitraum von insgesamt 1 Monat fortgesetzt. In der letzten Follow-up-Untersuchung 6 Wochen nach Erstvorstellung sahen wir eine oberflächliche Hornhautnarbe (3 × 2 mm) ohne Hinweis auf Aktivität, der Visus war 0,8 mit eigener Korrektur.

## Diskussion

*Paecilomyces lilacinus* ist ein Schimmelpilz, dessen Ausbreitung Feuchtigkeit voraussetzt, sodass dieser Pilz v. a. in Gemüse, Obst, Erde oder Gräsern zu finden ist [1]. Trotz der weiten Verbreitung wurden weltweit bisher nur wenige Fälle von Mykosen des Menschen beschrieben.

Infektionen manifestieren sich mit 51 % bevorzugt an der Augenoberfläche, gefolgt von der Haut mit 35 % [2]. Die Wärmeintoleranz des Organismus mit optimalen Temperaturen für die Sporenbildung bei ca. 20–25 °C könnte die Ursache hierfür sein [2]. Begünstigt wird die Infektion v. a. durch topische oder systemische Immunsuppression [3, 4]. Weitere Risikofaktoren sind das Tragen von (weichen) Kontaktlinsen, eine chronische Keratopathie oder Augenoperationen in der Vorgeschichte [5, 6].

Symptome können allmähliches Auftreten von Schmerzen, Fremdkörpergefühl, Photophobie, Verschwommensehen und eine Visusminderung sein [7]. Eine häufig gesehene Besonderheit der *Paecilomyces*-Keratitiden ist die Präsentation mit einem von Stromaanteilen bedeckten tiefen Stromainfiltrat, ggf. mit einer endothelialen Plaque und einer floriden Vorderkammerreaktion mit oder ohne Hypopyon [5, 7]. Im weiteren Verlauf kann sich der Organismus auf das anteriore Stroma ausbreiten. Zum Keimnachweis war daher in den bislang beschriebenen Fällen eine tiefe Hornhautbiopsie nötig, aufgrund oberflächlicher Probenentnahmen resultierten häufig falsch negative Ergebnisse [5]. Im Gegensatz dazu konnte in unseren Fällen die Diagnose mittels oberflächlichem Scraping gestellt werden. Weitere Erstmanifestationen können z. B. eine Skleritis, noduläre Episkleritis, anteriore Uveitis oder Endophthalmitis sein [4, 8].

Die Eradikation des Keims gestaltete sich aufgrund der typischerweise multiplen Resistenzen gegen Antimykotika lange Zeit sehr schwierig. Als schlecht wirksam zeigte sich v. a. Amphotericin (sowohl intravenös wie auch intrakameral oder topisch) [7, 9]. Auch lokal appliziertes Natamycin, Ketoconazol und Itraconazol konnten keine zufriedenstellenden Therapieergebnisse erzielen [7]. Mit der Einführung von Voriconazol verbesserte sich die Prognose erheblich, eine Operation bis hin zur perforierenden Keratoplastik oder Enukleation war dennoch häufig nötig [5–7, 10]. Entsprechend diesen Beobachtungen zeigte sich der Erreger auch in unseren Fällen sensibel gegen Voriconazol und resistent gegen Amphotericin.

Der günstige Verlauf der beiden hier beschriebenen Fälle könnte auf den raschen Erregernachweis und das zügige Einleiten einer antimykotischen Therapie, bevor sich der Schimmelpilz in die Tiefe ausbreiten konnte, zurückzuführen sein.

## Fazit

- Entgegen der häufig beschriebenen Manifestation der mykotischen *Paecilomyces*-Infektion im hinteren Hornhautstroma sahen wir 2 Fälle mit oberflächlichen Hornhautinfiltraten.
- Es wird die Bedeutung eines zeitnahen Hornhautscrapings bei Kontaktlinsenträgern mit oberflächlichen Hornhautinfiltraten oder verzögerter Wundheilung/Epithelirregularitäten deutlich.
- Durch das prompte Einleiten einer antimykotischen Therapie mit Voriconazol können so eine Ausbreitung in das tiefe Stroma und eine intraokuläre Invasion verhindert werden.
